# Unlocking the potentials of nitrate transporters at improving plant nitrogen use efficiency

**DOI:** 10.3389/fpls.2023.1074839

**Published:** 2023-02-21

**Authors:** Oluwaseun Olayemi Aluko, Surya Kant, Oluwafemi Michael Adedire, Chuanzong Li, Guang Yuan, Haobao Liu, Qian Wang

**Affiliations:** ^1^ Tobacco Research Institute of Chinese Academy of Agricultural Sciences, Qingdao, China; ^2^ Graduate School of Chinese Academy of Agricultural Sciences, Beijing, China; ^3^ Agriculture Victoria, Grains Innovation Park, Horsham, VIC, Australia; ^4^ School of Applied Systems Biology, La Trobe University, Bundoora, VIC, Australia; ^5^ School of Agriculture, Federal College of Agriculture, Ibadan, Nigeria

**Keywords:** nitrate transporters, nitrate uptake, nitrate transport and signaling, nitrate remobilization, nitrogen use efficiency, environmental stress

## Abstract

Nitrate ( ${\text{NO}}_{3^{-}}$ ) transporters have been identified as the primary targets involved in plant nitrogen (N) uptake, transport, assimilation, and remobilization, all of which are key determinants of nitrogen use efficiency (NUE). However, less attention has been directed toward the influence of plant nutrients and environmental cues on the expression and activities of ${\text{NO}}_{3^{-}}$ transporters. To better understand how these transporters function in improving plant NUE, this review critically examined the roles of ${\text{NO}}_{3^{-}}$ transporters in N uptake, transport, and distribution processes. It also described their influence on crop productivity and NUE, especially when co-expressed with other transcription factors, and discussed these transporters’ functional roles in helping plants cope with adverse environmental conditions. We equally established the possible impacts of ${\text{NO}}_{3^{-}}$ transporters on the uptake and utilization efficiency of other plant nutrients while suggesting possible strategic approaches to improving NUE in plants. Understanding the specificity of these determinants is crucial to achieving better N utilization efficiency in crops within a given environment.

## 1 Introduction

Nitrogen (N) is an essential element required for plant growth and overall yield; hence, the demand and use of N**-**based chemical fertilizers have consistently increased over the years. Approximately 60**-**70% of the applied N fertilizers are lost to the environment (Mohanty et al., 2020), causing severe environmental havoc such as pollution, global warming, biodiversity loss, and major plant physiological disorders. Since the increasing rate of N application is becoming increasingly alarming, minimizing fertilizer use while maintaining a high crop yield would be imperative. Thus, improving plants’ nitrogen use efficiency (NUE) is one of the inherent ways of overcoming these crises associated with crop production. Efficient N utilization is a critical factor in crop yield improvement, and research has shown that over 1.0 billion US dollars might be saved with a one percent NUE increment (Kant et al., 2011a).

Crop NUE is the measure of seed yield, grain, or fruit corresponding to a unit of soil N supplied, depending on the individual species of plant. NUE can also be expressed in terms of N uptake efficiency (NUpE), N transport efficiency (NTE), N remobilization efficiency (NRE), and N utilization (assimilation) efficiency (NUtE) (Bharati and Mandal, 2019), all of which are key determinant factors of NUE in plants. N is made available to plants in organic and inorganic forms; nitrate ( ${\text{NO}}_{3^{-}}$ ) and ammonium. Due to the mobility nature of ${\text{NO}}_{3^{-}}$ , it gets easily leached; thus, its availability to plants becomes limiting (Jin et al., 2015). ${\text{NO}}_{3^{-}}$ functions as a signaling molecule, inducing the expression of NO_3_^-^-related genes involved in its uptake, transport, assimilation, vegetative and reproductive development. Plants take up ${\text{NO}}_{3^{-}}$ from the root, assimilate ${\text{NO}}_{3^{-}}$ , and subsequently transport it to the shoot, where it can be remobilized to sink organs (Iqbal et al., 2020). ${\text{NO}}_{3^{-}}$ transporters are the main drivers involved in the uptake of ${\text{NO}}_{3^{-}}$ to the remobilization stage.

Indeed, several studies have discussed the relationship between ${\text{NO}}_{3^{-}}$ uptake transport activities in plants while addressing the mechanisms involved in transport, sensing, and signaling processes (Fan et al., 2017; Zuluaga and Sonnante, 2019; Vidal et al., 2020). Therefore, optimizing the activities of ${\text{NO}}_{3^{-}}$ transporters is a prerequisite for plants to utilize N supplies. Some studies have elucidated the functional roles of these ${\text{NO}}_{3^{-}}$ transporters in plant NUE improvement. However, less is known about the influence of essential nutrients and environmental cues on the expression and activities of ${\text{NO}}_{3^{-}}$ transporters. To better understand the extent to which these transporters can function in improving plant NUE, an illustration of their response to changes in plant environmental cues, including salinity, pathogenic and drought stress, and contamination from heavy metals, becomes expedient. Even if these conditions are being optimized, it is crucial to explore the possible aftermath effect of these ${\text{NO}}_{3^{-}}$ transporters on the efficiency of other plant nutrient elements and related factors. These necessities ignite a few questions: 1) Does stress affect ${\text{NO}}_{3^{-}}$ transporter activities directly or indirectly? and 2) Do the activities of these ${\text{NO}}_{3^{-}}$ transporters exert a positive or negative effect on the uptake of other nutrients? To resolve these issues, this review critically summarized the roles of ${\text{NO}}_{3^{-}}$ transporters in N uptake, transport, and distribution processes and their functions in crop productivity and NUE, especially when coexpressed with other transcription factors. This review focuses on the functional roles of these nitrate transporters in assisting plants in adverse environmental conditions. We also discussed the impact of these ${\text{NO}}_{3^{-}}$ transporters on the uptake and utilization efficiency of other plant nutrients while describing possible strategic approaches to improving NUE in plants. The contribution of nitrate transporters in nitrate and auxin crosstalk for root growth and NUE is also reviewed. Understanding the specificity of all these factors is crucial for better N utilization efficiency of crops.

## 2 Nitrate uptake and transport systems

Most agricultural fields, especially, those used for commercial crop production, are ${\text{NO}}_{3^{-}}$ deficient with significant spatiotemporal fluctuations, inhibiting N utilization (Kant, 2018). Plants have evolved two major ${\text{NO}}_{3^{-}}$ uptake mechanisms to survive. The first is the low**-**affinity transport system (LATS), which facilitates nitrate uptake under high soil-N (millimolar concentration; > 0.5 mM), while the other is the high**-**affinity transport system (HATS), which drives nitrate under insufficient soil**-**N (micromolar range) (Léran et al., 2014; Iqbal et al., 2020; Raddatz et al., 2020). Four families of ${\text{NO}}_{3^{-}}$ transporters have been widely known to participate in plant nitrate uptake and transport: nitrate transporter 1/or peptide transporter NPF (NRT1), nitrate transporter 2/nitrate-nitrite-porter NRT2/NNP, slow anion channel**-**associated homologs (SLAC/SLAH), and chloride channel (CLC) (Tsay et al., 1993; Bergsdorf et al., 2009; Maierhofer et al., 2014; Von Wittgenstein et al., 2014). Among them, NPF (NRT1) and NRT2 and homologs have been identified as the major channels actively involved in root nitrate uptake and long**-**distance transport between and within plant organs (Hsu and Tsay, 2013; Wang et al., 2021b). In this review, proteins or genes void of prefixes connote Arabidopsis plant species.

**Figure 1:**
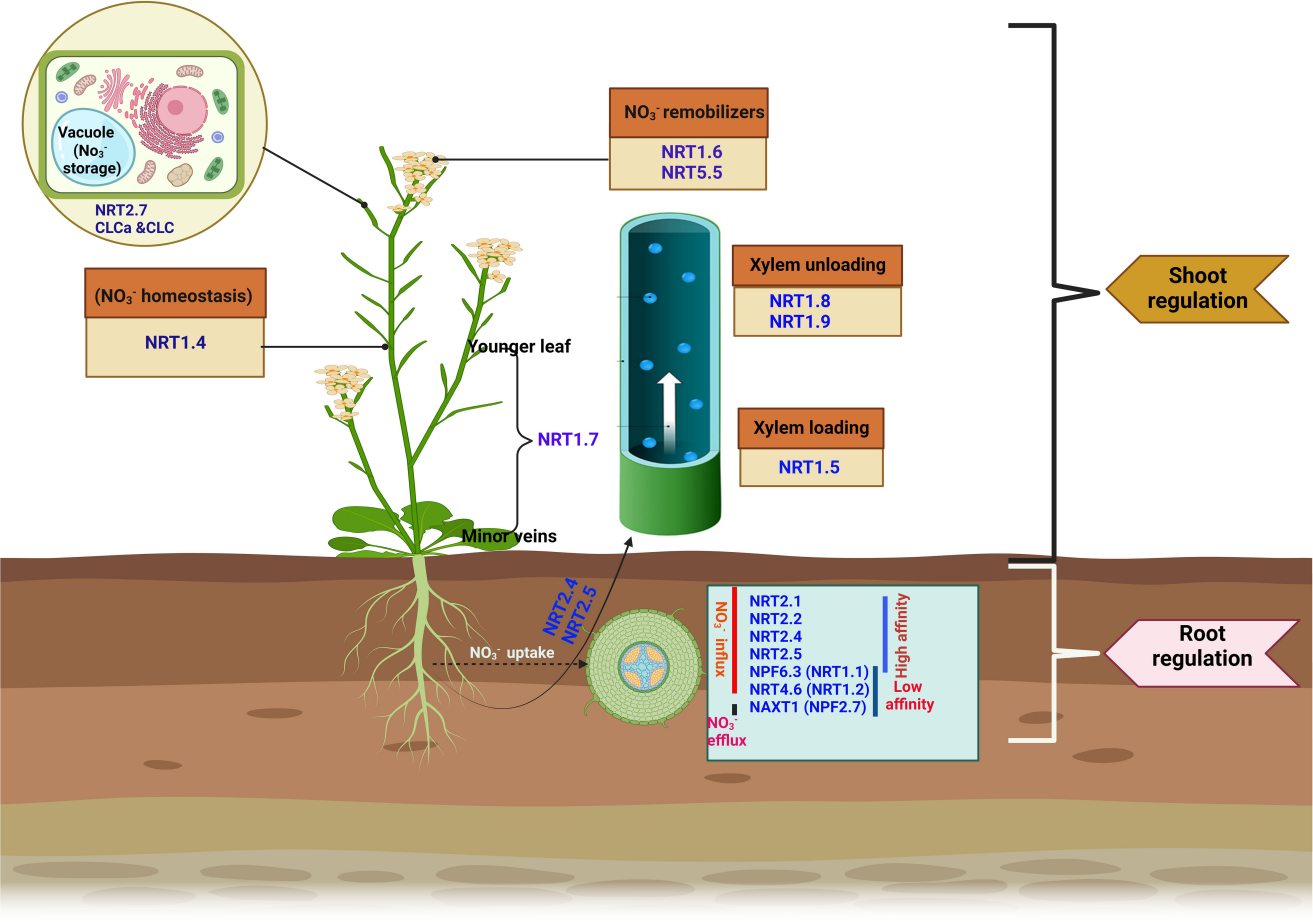
Key nitrate transporters involved in nitrate uptake, transport, and remobilization in plants. Nitrate transporters involved in ${\text{NO}}_{3^{-}}$ acquisition from the root include *NRT2.1*, *NRT2.2*, *NPF4.6* (*NRT1.2*), *NRT2.4*, *NRT2.5*, and *NPF6.3* (*NRT1.1*). *NPF2.7* performs the ${\text{NO}}_{3^{-}}$ efflux function. In addition to the uptake function, *NRT2.4* and *NRT2.5* facilitates root-to-shoot ${\text{NO}}_{3^{-}}$ transport. *NRT1.5* is responsible for xylem loading, while *NRT1.8* and *NRT1.9* functions to unload ${\text{NO}}_{3^{-}}$ from the xylem. *NRT1.4* regulates ${\text{NO}}_{3^{-}}$ homeostasis, and the expression of *NRT1.7* in the phloem of the minor vein promotes nitrate remobilization from mature to younger leaves. At shoot, *NRT1.6* and *NPF5.5* act as a ${\text{NO}}_{3^{-}}$ remobilizer, remobilizing ${\text{NO}}_{3^{-}}$ in the embryo. *NRT2.7* enhances ${\text{NO}}_{3^{-}}$ storage in the seed vacuole.

Phylogenetic studies revealed that the NPF family comprises 53 identified Arabidopsis genes, and over 130 genes exist in higher plants (Zhang et al., 2020). Generally, NPF transporter genes have low affinity for ${\text{NO}}_{3^{-}}$ , except for Chlorate resistant 1/nitrate transporter 1 (*CHL1* **/** *NRT1.1*), also called *NPF6.3*, a dual**-**affinity nitrate transporter that operates as both a low**-** and high-affinity transporter (Liu and Tsay, 2003). The regulatory mechanism involved in the dual**-**affinity system enables the rapid switch between these two affinity modes. Under a low external supply of ${\text{NO}}_{3^{-}}$ , *NPF6.3* (*CHL1* **/** *NRT1.1*) functions as a high**-**affinity ${\text{NO}}_{3^{-}}$ transporter and is phosphorylated, whereas it becomes dephosphorylated under a high ${\text{NO}}_{3^{-}}$ supply to perform a low**-**affinity transporter role (Liu and Tsay, 2003; Noguero et al., 2018). Thus, the affinity of the *NPF6.3* transporter for ${\text{NO}}_{3^{-}}$ uptake depends on the phosphorylation state at the T101 residue, which is subject to the status of N in the medium *NPF6.3* (*CHL1*/*NRT1.1*) is expressed in various plant tissues, including younger leaves, flower buds, and roots, where it participates in root ${\text{NO}}_{3^{-}}$ uptake and translocation (Noguero et al., 2018). In addition to *NPF6.3* (*CHL1*/*NRT1.1*), *NPF4.6* (*NRT1.2*) and *NPF2.7* (*NAXT1*) are the two putative NPF genes that coordinate ${\text{NO}}_{3^{-}}$ influx and efflux in plant roots, respectively (**Figure 1**). *NPF4.6* (*NRT1.2*) is primarily expressed at the root tip where it takes up ${\text{NO}}_{3^{-}}$ (Huang et al., 1999), whereas *NPF2.7* (*NAXT1*), is expressed in the root zone but in the cortex, performs ${\text{NO}}_{3^{-}}$ **-**efflux functions (Segonzac et al., 2007). A considerable amount of NRT1 family members have been identified in other crops, including wheat (*Triticum aestivum*) (Kumar et al., 2022), rice (*Oryza sativa*) (Yang et al., 2020), cucumber (*Cucumis sativus*) (Migocka et al., 2013), potato (*Solanum tuberosum*) (Zhang et al., 2021a), and apple (*Malus × domestica Borkh*.) (Wang et al., 2018b), with their unique expression at either the root or shoot of plants. The expression pattern of these transporters is a clear indication of their active involvement in uptake and long-distance ${\text{NO}}_{3^{-}}$ transport.

Unlike the NRT1 family, NRT2 family members are high**-**affinity ${\text{NO}}_{3^{-}}$ transporters (HATs). There are eight identified NRT2 family members, of which seven have been characterized (Von Wittgenstein et al., 2014). Four (*NRT2.1*, *NRT2.2*, *NRT2.4*, and *NRT2.5*) out of the seven characterized NRT2 transporters have been actively involved in the influx of ${\text{NO}}_{3^{-}}$ into Arabidopsis root cells (O’Brien et al., 2016). Detailed functions of these transporters in uptake of ${\text{NO}}_{3}^{–}$ are presented in (**Figure 1**).

Nitrate transporters are the major channels mediating root-to-shoot ${\text{NO}}_{3^{-}}$ transport. Transport is predominantly mediated by NRT1 and NRT2 transporters, such as *NPF7.3* (*NRT1.5*), *NPF7.2* (*NRT1.8*), *NPF2.3*, and *NPF2.9* (*NRT1.9*). *NPF7.3* (*NRT1.5*) is expressed in pericycle cells, where it facilitates xylem loading of ${\text{NO}}_{3^{-}}$ (**Figure 1**). Knockout *nrt1.5* mutant plants had reduced amounts of ${\text{NO}}_{3^{-}}$ translocated from the roots to the shoots. However, when *NRT1.5* was reduced in *nrt1.5*, no ${\text{NO}}_{3^{-}}$ translocation defect was observed, suggesting the existence of another mechanism facilitating nitrate xylem loading (Lin et al., 2008). The low-affinity nitrate transporters *NRT1.8* and *NRT1.9* perform similar roles of unloading ${\text{NO}}_{3^{-}}$ from the xylem (**Figure 1**), consequently reducing ${\text{NO}}_{3^{-}}$ concentration within the xylem. Knockout mutants of such transporters *(NRT1.8* and *NRT 1.9*) exhibited increased amounts of ${\text{NO}}_{3^{-}}$ in the xylem and, by implication, accelerated root-shoot transport of nitrate (Li et al., 2010; Wang and Tsay, 2011). In addition, the uptake and transport function of the NRT1 and NRT2 homologs have also been revealed in rice (*OsNRT1.1B* and *OsNRT2.3*, respectively) (Tang et al., 2012; Hu et al., 2015; Fan et al., 2017), and tomato, *LeNRT2.3* (Fu et al., 2015).

While ${\text{NO}}_{3^{-}}$ is relocated to the shoot, a larger proportion of N is delivered to the sink organs (e.g., seeds, fruits, roots, and younger leaves), especially for the anabolic development of new tissues, prioritized by the growth stage or physiological condition of individual plants, a process called N remobilization (Snyder and Tegeder, 2021). NRT1.4, localized in the leaf petiole, regulates ${\text{NO}}_{3^{-}}$ accumulation within the petiole while maintaining the homeostasis of available ${\text{NO}}_{3^{-}}$ between the leaf lamina and petiole (**Figure 1**). The *nrt1.4* mutant had a low ${\text{NO}}_{3^{-}}$ content in its petiole, a major ${\text{NO}}_{3^{-}}$ storage organ, indicating the involvement of *NRT1.4* in nitrate homeostasis and leaf development (Chiu et al., 2004). Another ${\text{NO}}_{3^{-}}$ transporter, *NRT1.7*, predominantly expressed in the phloem of minor veins, enhances nitrate relocation from older to younger leaves (**Figure 1**) (Fan et al., 2009). However, the extent of ${\text{NO}}_{3^{-}}$ transfer and the proportion of ${\text{NO}}_{3^{-}}$ remobilized to the sink organ remain unclear. ${\text{NO}}_{3^{-}}$ storage in seeds is mediated by specific ${\text{NO}}_{3^{-}}$ transporters that remobilize ${\text{NO}}_{3^{-}}$ into embryos during seed formation. The expression of *NRT1.6* within the host embryo and seed coat demonstrates a potential role of this transporter in mediating embryonic ${\text{NO}}_{3^{-}}$ relocation at the reproductive phase of the parent plant (**Figure 1**) (Almagro et al., 2008). Similar to *NRT1.6*, *NPF5.5* also mediates ${\text{NO}}_{3^{-}}$ transport into the embryo (**Figure 1**) (Léran et al., 2015; Iqbal et al., 2020). *NRT2.7*, a high-affinity ${\text{NO}}_{3^{-}}$ transporter in the tonoplast, plays specific ${\text{NO}}_{3^{-}}$ storage roles in the seed vacuole (Chopin et al., 2007). In the tonoplast, CLCa and CLCb were observed to perform a similar localization pattern, where they also participate in ${\text{NO}}_{3^{-}}$ storage (Von Der Fecht-Bartenbach et al., 2010). While ${\text{NO}}_{3^{-}}$ accumulation in seed vacuoles has been well documented, relatively less is understood about the characterization of transporter genes involved in ${\text{NO}}_{3^{-}}$ efflux out of the vacuole. An in-depth understanding of the specificity of these N transporters, from chronological studies, is the first step toward exploiting and optimizing NUE in plants.

## 3 Nitrogen assimilation in relation to NUE

For efficient ${\text{NO}}_{3^{-}}$ assimilation, a larger proportion of ${\text{NO}}_{3^{-}}$ assimilated after root uptake is diverted back to the cytosol, where it is converted to nitrite by nitrate reductase (NR). The nitrite obtained is relocated to plastids for subsequent reduction. At this stage, nitrite is converted to ammonium ( ${\text{NH}}_{4}^{+}$ ) by the nitrite-reducing enzyme nitrite reductase (NiR) and then finally incorporated as an amino acid through the glutamine synthetase (GS) and glutamate synthase (GOGAT) cycle (Wilkinson and Crawford, 1993; Li et al., 2017a). Nitrogenous compounds incorporated *via* glutamine (free amino acid) and glutamate serve as a major checkpoint for regulating N utilization efficiency and are further enhanced by the synergetic expression of NR and ${\text{NO}}_{3^{-}}$ transporters (Li et al., 2020; Snyder and Tegeder, 2021). However, a recent study opined an improved grain yield and NUE on concurrent coexpression of *OsNRT1.1B* and indica *OsNR2*, indicating the positive regulatory roles of *OsNR2* and *OsNRT1.1B* in uptake of N in rice (Gao et al., 2019b).

The two functionally similar forms of GS, cytosolic GS1, and plastidic GS2, encoded by single or multiple gene families, have been reported to significantly influence N assimilation (Miflin and Habash, 2002). While cytosolic GS1 facilitates root N reassimilation and remobilization during protein turnover, GS2 isoforms primarily assimilate ${\text{NH}}_{4}^{+}$ produced during chloroplast photorespiration (Ferreira et al., 2019). Although GS1 is responsible for ${\text{NH}}_{4}^{+}$ reassimilation, some GS family members drive N assimilation when ${\text{NO}}_{3^{-}}$ is abundant. A good example is *GLN1*;*2* in Arabidopsis, which drives N assimilation when ${\text{NO}}_{3^{-}}$ is abundant, compared to the *gln1*;*2* mutant, which exhibits reduced GS activity, rosette biomass, and higher ${\text{NH}}_{4}^{+}$ concentration under such conditions. Due to the principal roles of GS in N assimilation, specific focus has been directed toward overexpressing GS family members to improve N assimilation in different plant species, such as *Triticum aestivum* (Hu et al., 2018), and *Oryza sativa* (Bao et al., 2014).

Despite the fundamental roles of GS in improving ${\text{NH}}_{4}^{+}$ assimilation, seed yield, and NUE (Hu et al., 2018; Gao et al., 2019a), attempts to improve NUE by overexpressing *GS1* have yielded inconsistent results (Check **Table 1** for details). For instance, *TaGS2-2Ab*-overexpressing lines in wheat had increased spike number, seed yield, and NUE under poor and rich N supply compared to their wild type, due to an increased root N uptake and remobilization capacity (Hu et al., 2018). Following a similar trend, overexpressing *HvGS1-1* using its promoter confers improved grain yield and NUE on barley subjected to low and high N conditions (Gao et al., 2019a). In contrast, Bao et al. (2014) opined a drastic reduction in fresh and dry weight of *OsGS1*;*1*- and *OsGS1*;*2*-overexpressing lines in rice seedlings, with a further poor growth phenotype at the tillering and heading stages under limited and sufficient N conditions. The results suggest that the GS-overexpressing lines and plant biomass are negatively correlated. Further research is required to understand the underlying mechanisms of GS activity to improve NUE in plants.

**Table 1:** Nitrogen assimilatory genes involved in nitrogen use efficiency.

S/N	Genes	Host species	Transgenic approach	Effects	References
1	*OsGS1;2*	Rice	Overexpression	• Improves N utilization efficiency	(Brauer et al., 2011)
				• Enhances N harvest index	
				• May not lead to less N input under field condition	
2	*GS1;1, GS1;2*	Rice	Overexpression	Poor yield and growth phenotypes under different N conditions.	(Bao et al., 2014)
3	*OsNADH-GOGAT*	Rice	Overexpression	Enhances N utilization and grain filling	(Yamaya et al., 2002)
4	*OsAlaAT*	Rice	Overexpression	Increases nitrate uptake efficiency, tiller number, and grain yield	(Shrawat et al., 2008; Beatty et al., 2009)
5	*OsAAT1-3*	Rice	Overexpression	Increases protein and amino acids in seeds	(Zhou et al., 2009)
6	*ASN1*	Arabidopsis	Overexpression	• Increases seedlings’ tolerance to low N supply	(Lam et al., 2003)
				• Improves protein content in the seeds	
7	*HvGS1.1*	Barley	Cisgenic expression	Increased grain yields and NUE	(Gao et al., 2019a)
8	*TaGS2-2Ab*	Wheat	Transgenic expression	Improves grain yields and NUE under different N conditions	(Hu et al., 2018)
9	*ZmGln1-3*/*ZmGln1-4*	Maize	Mutation	Exhibits reduced kernel size and number	(Martin et al., 2006)

Unlike GS, relatively few studies have addressed alterations in the expression of genes encoding NADH-dependent GOGAT (a key enzyme in N assimilation) and plastid**-**localized ferredoxin-dependent (Fd-GOGAT) (Good et al., 2004; Xu et al., 2012). The two kinds of GOGAT differ in their electron donor specificity. Fd**-**GOGAT is predominantly involved in the reassimilation of photorespiratory ${\text{NH}}_{4}^{+}$ . In contrast, NADH-GOGAT participates in the assimilation of non**-**photorespiratory ${\text{NH}}_{4}^{+}$ and the synthesis of glutamate needed for plant development (Lee et al., 2020). Many attempts have been devoted to studies on the fundamental roles of both NADH-GOGAT and Fd-GOGAT in the growth and seed development of Arabidopsis (Somerville and Ogren, 1980), *Hordeum vulgare* L. (Kendall et al., 1986), and *Oryza sativa* (Zeng et al., 2017). However, few research studies have altered the genetic expression of GOGAT to promote seed yield and NUE, while those that focused on NADH-GOGAT had rather limiting outcomes. For example, overexpression of *ZmNADH* **-** *GOGAT* in maize confers drastic reduction on shoot biomass with no considerable alterations in kernel yield when N is abundant (Cañas et al., 2020). Meanwhile, the overexpression lines of *OsNADH* **-** *GOGAT* resulted in an increase in rice grain weight under limited N (Yamaya et al., 2002). Interestingly, Lee et al. (2020) recently revealed that the synergetic expression of *OsNADH* **-** *GOGAT1* and *OsAMT1;2* confers an increase in NUE under both high and low N supply. While transgenic lines had improved seed protein levels without any yield alteration under N-sufficient conditions, seed quality and overall yield increased under N starvation. These observations imply that the combined expression of N-transporters and GOGAT improves N uptake, N assimilation, and NUE rather than the negative effect of the expression of AMT or GOGAT alone. Consequently, understanding the factors involved in the synergetic expression of ${\text{NO}}_{3^{-}}$ transporters and GOGAT under rich and poor N conditions in plants is imperative to augment NUE.

## 4 Nitrate sensing and signaling

In addition to its nutritional roles, ${\text{NO}}_{3^{-}}$ functions as a major signaling element regulating several plant physiological processes, such as leaf expansion (Walch‐Liu et al., 2000), induction of root architectural changes (Walch‐Liu and Forde, 2008), regulation of root development, and regulation of floral induction (Marín et al., 2011). The first step in signaling is through external nitrate perception by the dual affinity ${\text{NO}}_{3^{-}}$ transporter *NPF6.3* (*NRT1.1*), induced immediately after ${\text{NO}}_{3^{-}}$ treatment. *NRT1.1* switches between two states of nitrate conditions (low and high ${\text{NO}}_{3^{-}}$ conditions) (Wang et al., 1998; Bouguyon et al., 2015; Hu et al., 2015).

### 4.1 Roles of transcription factors in N use regulation

Several transcription factors (TFs) have been reported to play critical roles in NUE regulation by modulating the expression of ${\text{NO}}_{3}^{–}$ responsive genes. Detailed functions of TFs involved in NUE improvements are outlined in **Table 2**. DNA binding with one finger (*Dof1*) TFs increases N use in plants. The transgenic expression *of ZmDof1* in *A. thaliana* (Yanagisawa et al., 2004), *TaDof1* in wheat (Hasnain et al., 2020), *ZmDof1* in rice (Kurai et al., 2011), wheat and sorghum (Peña et al., 2017) improve N assimilation and plant growth under N starvation.

**Table 2:** Transcription factors (Tfs) involved plant nitrogen use efficiency.

Family	Tfs	Host species	Transgenic approach	Summary of findings	Reference
**MADS-box**	*ANR1*	Arabidopsis	Overexpression	Rapid early seedling developments	(Gan et al., 2012)
	*AGL21*	Arabidopsis	Overexpression	Increases lateral root (LR) density and length	(Yu et al., 2014)
	*OsMADS25*	Rice	Overexpression	• Promotes nitrate accumulation and upregulates other ${\text{NO}}_{3^{-}}$ responsive genes • Positively regulates primary and LR development	(Yu et al., 2015)
	*OsMADS57*	Rice	Overexpression	• Regulates nitrate root-to-shoot transport • Upregulates *OsNRT2.1*/*2.2*/*2.4* and *OsNRT2.3a.*	(Huang et al., 2019)
	*CmANR1*	Arabidopsis	Overexpression	• Improves lateral root growth and development under moderate ${\text{NO}}_{3^{-}}$ regime • 7.5%-116.2% increase in root auxin level	(Sun et al., 2018)
	*ZmTMM1*	Arabidopsis	Overexpression	Increases NR, GS, and PEPC activity and LR elongation	(Liu et al., 2020)
**Dof**	*ZmDof1*	Rice	Constitutive expression	Improves N assimilation and growth under N-deficient condition	(Yanagisawa et al., 2004; Kurai et al., 2011)
	*Dof1(Dof1.7)*	Tobacco	Overexpression	Increases plant length, total protein, and N assimilation under low N	(Wang et al., 2013)
	*ZmDof1*	Wheat and Sorghum	Constitutive expression	• Negatively affects photosynthesis, plant height, and biomass under poor-N • Reduces the expression of photosynthetic-regulatory genes	(Peña et al., 2017)
	*TaDof1*	Wheat	Overexpression	• Regulates Carbon and N metabolism under N-limiting conditions. • Improves different agronomic traits	(Hasnain et al., 2020)
**bZIP**	*TGA4*	Arabidopsis	Overexpression	• Alleviates N-starvation • Enhances nitrate transport and assimilation capacity.	(Zhong et al., 2015)
	*TabZIP60*	Wheat	Downregulation (RNAi)	• Stimulates lateral root branching, spike number and increases N uptake •; Accelerates NADH-dependent glutamate synthase (NA–H - GOGAT) activity • Improves grain yield by more than 25% under field-based conditions	(Yang et al., 2019)
	*HY5/HYH*	Arabidopsis	Knockout	Upregulates *NRT1.1* and improves N-uptake	(Jonassen et al., 2009)
	*TGA1/4*	Arabidopsis	Mutation based	• Increases the expression of *NRT1.1*, *NRT2.1*, represses *NIA2* • Decreases LR growth and root hair density	(Canales et al., 2017)
**NLP**	*OsNLP1*	Rice	Overexpression	Increases plant growth, yield, and NUE under diverse N supplies.	(Alfatih et al., 2020)
	*OsNLP4*	Rice	Overexpression	Improves plant biomass, yield, and NUE under moderate N	(Wang et al., 2021a)
	*ZmNLP6 and ZmNLP8*	Arabidopsis	Overexpression	• Increases biomass and yield by 15% and 45% under low N • Contributes to NUE	(Cao et al., 2017)
	*ZmNLP5*	Maize	Mutation based	• Decreases in root NO_3_- accumulation • Reduces ear, seed kernels, and leaves N contents • Suppresses shoot ${\text{NH}}_{4}^{+}$ content.	(Ge et al., 2020)
	*NLP7*	Arabidopsis	Overexpression	Increases plant growth under low and high-N conditions	(Yu et al., 2016)
**MYB**	*OsMYB305*	Rice	Overexpression	• Improves nitrate uptake, N assimilation, and growth • Improve NUE	(Wang et al., 2020a)
	*SiMYB3*	Arabidopsis/and rice	Overexpression	• Improves seed N, grain weight, total N, and root growth • Upregulates *OsNRT2.1*, *OsNRT2.2*, *OsNiR2*, and *OsNAR2.1*	(Ge et al., 2019)
	*MYB59*	Arabidopsis	Mutation based	• Reduces K^+^/ ${\text{NO}}_{3^{-}}$ root-to-shoot transport • Represses *NRT1.1* expression.	(Du et al., 2019)
**Lateral organ boundary domain (LBD)**	*LBD37* *LBD38* *LBD39*	Arabidopsis	Overexpression	Downregulates several N-related genes	(Rubin et al., 2009)
**Zinc-finger proteins**	*GATA4*	Arabidopsis	Downregulation	• Higher shoot biomass and root hair density • Fewer LRs, and shorter PRs	(Shin et al., 2017)
**NAC**	*TaNAC2-5A*	Wheat	Overexpression	• Increases tiller number and dry weight under low ${\text{NO}}_{3^{-}}$ starvation • Improved grain and shoot N, harvest index, and grain yield	(He et al., 2015)
	*NAM-B1*	wheat	Downregulation (RNAi)	• Enhances leaf N to grain remobilization	(Uauy et al., 2006)
**NF-Y**	*TaNFYA-B1*	Wheat	Overexpression	Increases root growth, N uptake, and grain yield	(Qu et al., 2015)
**ZYF**	*TaZFP593;l*	Wheat	Overexpression	• Improves root system architecture, N uptake, and grain yield under low N	(Chen et al., 2017)

PR, Primary roots; PEPC, Phosphoenolpyruvate carboxylas.

The key regulators of nitrate assimilatory genes, teosinte branched1-cycloidea-proliferating cell factor1-20 (TCP20) and NIN-like protein (NLP), NLP6 and NLP7 interact with each other under N sufficient and N–starved condition to control ${\text{NO}}_{3^{-}}$ response to root growth (Guan et al., 2017), a strong indication of NLP’s involvement in ${\text{NO}}_{3^{-}}$ signaling-related responses. Moreover, overexpression of *NLP7* results in positive regulation of key nitrate metabolites, total N contents, ${\text{NO}}_{3^{-}}$ uptake, and signaling-related genes while improving plant biomass under poor and rich N conditions in Arabidopsis. This peculiar function suggests *NLP7* as a master regulator of the primary nitrate response and its importance in plant N use (Yu et al., 2016). Further research on NLP family members reveals that overexpressing *ZmNLP6* and *ZmNLP8* in Arabidopsis replaces the roles of *NLP7* in ${\text{NO}}_{3^{-}}$ signaling, and metabolism (Cao et al., 2017). In a recent study by Wu et al. (2021), overexpression of *OsNLP4* in rice increased grain yield and NUE by 30% and 47%, respectively, under moderate N conditions. Contrary to NLP, three lateral organ boundary domain TFs (LBD37, LBD38, and LBD39) negatively regulate nitrate uptake and assimilatory genes, and thus could be candidates for improving NUE in plants (Rubin et al., 2009).

A putative MADS-box TF, *ANR1*, associated with lateral root growth and elongation (Zhang and Forde, 1998), functions as a downstream regulator of NRT1 in response to nitrate (Remans et al., 2006). In addition, *AGL21* (AGAMOUS-Like 21) functions in lateral root initiation and growth by regulating auxin biosynthetic genes under N**-**deficient conditions (Yu et al., 2014). Although, other TFs efficiently utilizing N in Arabidopsis and cereal crops (especially rice) have been identified, the focus on identifying these genes in other crops has been minimal.

### 4.2 Nitrate-induced MicroRNA regulation

MicroRNAs (miRNAs) are small noncoding RNAs containing approximately 20**-**24 nucleotides with diverse regulatory potentials (Zhou et al., 2020). Studies have shown that miRNAs regulate gene expression pathways related to plant growth and developmental processes in response to nitrate (check **Table 3** for further details) (Zuluaga and Sonnante, 2019). The upregulation or downregulation of miRNAs primarily anchors on their capacity to regulate key target N-related genes (Zhao et al., 2011). Research has also examined the crucial roles of miR169 family members in cereal crops. A drastic reduction in the expression level of miR169 was observed in N-starved maize (Zhao et al., 2012) and wheat (Qu et al., 2015), upregulating *TaNFYA-Bi* under such conditions. Despite the numerous miRNA-related NUE phenotypes identified, little is known about the regulatory mechanisms involved. Thus, further research is required to fully understand how N use can be optimized in plants.

**Table 3:** MicroRNAs involved in nitrogen use efficiency.

S/N	Genes	Host species	Transgenic approach	Summary of findings	Reference
1	*OsmiR393*	Rice	Mutation	Represses N-promoted tillering	(Li et al., 2016b)
2	*Osa-miR528*	Creeping Bentgrass	Overexpression	Increases total N, chlorophyll synthesis, and biomass accumulation	(Yuan et al., 2015)
3	*TaMIR444a*	Tobacco	Overexpression	Increases N uptake and plant biomass under N- limitation	(Gao et al., 2016)
4	*TaMIR2275*	Tobacco	Overexpression	Improves N and biomass accumulation under N starvation.	(Qiao et al., 2018)
5	*RDD1*	Rice	Overexpression	Increases N-uptake and grain yield under low N	(Iwamoto and Tagiri, 2016)

## 5 Nitrate transporters involved in NUE and yield improvement

Nitrate transporters have been shown to play diverse NUE and yield improvement roles in plants (Check **Table 4** for details). In Arabidopsis, *NRT1.1* transgenic lines habouring *Cauliflower Mosaic Virus* (CaMV) 35S promoter were observed to increase the uptake of ${\text{NO}}_{3}^{-}$ , however, this did not necessarily improve seed yield (Liu et al., 1999). In contrast, the expression of the *NRT1.1* homolog *OsNRT1.1B* driven by the CaMV-35S promoter or its native promoter increased NUE and grain yield in rice. The key regulatory roles in ${\text{NO}}_{3^{-}}$ nitrate signaling, absorption, and assimilation enable *OsNRT1.1B* to be a major contributor of rice NUE (Hu et al., 2015). Although, the crucial roles of *OsNRT1.1B* in NUE and yield improvement have been well studied, the underlying regulatory mechanism has not been elucidated. Similar to *OsNRT1.1B*, overexpression of the spliced form *OsNRT1.1A* also exhibits an approximately 50% grain yield and NUE increase, coupled with shortened maturation times (Wang et al., 2018c). The observations of this latter experiment could be successfully used to develop early maturing and high-yielding varieties in some other crops. The elevated expression of *OsNPF8.20* (*OsPTR9*) leads to increased ${\text{NH}}_{4}^{+}$ uptake, better root formation, and ultimately, an increased tiller and panicle number, indicating that *OsNPF8.20* improves grain yield and NUE in rice breeding (Fang et al., 2013). Similarly, *OsNPF7.20*-overexpressing lines exhibited a drastic increase in rice tiller number, fresh weight, dry weight, and grain yield. In contrast, an opposite effect was conferred on the RNA interference (Ri) lines and *osnpf7.2* mutant line under mixed nitrate supply (0.5-8 mM ${\text{NO}}_{3^{-}}$ ) (Wang et al., 2018a). In their experiment on the modification of ${\text{NO}}_{3^{-}}$ transporters in Arabidopsis and rice, Liu et al. (1999) and Hu et al. (2015) reported some discrepancies in the response of these plants to the modified transporters. This may be due to the tolerance and sensitivity of both crops to ${\text{NH}}_{4}^{+}$ and ${\text{NO}}_{3^{-}}$ . *Arabiodopsis* thrives under aerobic conditions where the ${\text{NO}}_{3^{-}}$ transport system is well optimized, whereas rice thrives best in anaerobic environments where the ${\text{NH}}_{4}^{+}$ transport system is optimized. Hence, manipulating ${\text{NO}}_{3^{-}}$ and ${\text{NH}}_{4}^{+}$ transporters for improved efficiency in Arabidopsis and rice, respectively, would generate little or no effect on their NUE. Several ${\text{NO}}_{3^{-}}$ transporter genes in plants whose expression and subcellular localization pattern greatly determine the gene’s function are essential in genetic manipulations of plant traits. As such, deep insight into the function of a gene and the environment to which plants are better adapted can encourage precise manipulation of NUE in crops. The influence of nitrate transporters on crop yield was also reported in tomatoes, where overexpression of *LeNRT2.3* improved ${\text{NO}}_{3^{-}}$ uptake, root-to-shoot ${\text{NO}}_{3^{-}}$ transport, plant biomass, and fruit weight (Fu et al., 2015).

**Table 4:** Nitrate transporter genes involved in plant nitrogen use efficiency.

S/N	Gene	Host plants	Expression pattern	Promoter region	Summary of findings	Reference
1	*OsNPF8.20* (*OsPTR9*)	Rice	Root tips, leaves, stems, and panicles	Ubi promoter	Increases ${\text{NH}}_{4}^{+}$ uptake, lateral root, and grain yield.	(Fang et al., 2013)
2	*OsNPF6.5* (*NRT1.1B*)	Rice	Root epidermis, root hairs, and vascular tissues	CaMV 35S or native promoter	Improves NUE and grain yield	(Hu et al., 2015)
3	*OsNPF8.9* (*OsNRT1.1a* and *OsNRT1.1b*)	Rice	Roots	Ubi promoter	• Increases shoot biomass under the hydroponic system	(Fan et al., 2016a)
					• Under low N conditions, *OsNRT1.1b* enhances N content and growth, but loss of function in *OsNRT1.1a*	
4	*OsNRT2.1*	Rice	Root, leaf sheaths, and leaf blades	Ubi and NAR2.1 promoter	• *pUbi*: *OsNRT2.1* exhibits decreased NUE	(Chen et al., 2016)
					• p*OsNAR2.1*:*OsNRT2.1* exhibits increased NUE	
5	*OsNPF7.3* (*OsPTR6*)	Rice	Roots and shoots	Ubi promoter	Improved growth under various N supplies but decreased NUE on excessive ${\text{NH}}_{4}^{+}$ supply	(Fan et al., 2014)
6	*OsNRT2.3a*	Rice	Culms	p35S:NRT2.3a	• *p35S*: *NRT2.3a* exhibits no improvement yield and NUE	(Fan et al., 2016b; Chen et al., 2020a)
				p35S:OsNAR2.1-p35S:OsNRT2.3a	• *p35S*:*OsNAR2.1*-*p35S*: *OsNRT2.3a* increases rice yield and NUE	
7	*OsNRT2.3b*	Rice	Phloem	CaMV 35S/Ubi promoter	• Increases the uptake of other mineral nutrients	(Fan et al., 2016a)
					• Improves grain yield and NUE by 40%	
8	*NRT1.7*	Arabidopsis, tobacco, and rice	Old leaves	NRT1.7 promoter (NRT1.7p::NC4N::3′)	• NO_3_- accumulation at the younger leaves	(Chen et al., 2020b)
					• Enhances NO_3_- remobilization to the sink,	
					• Improves plant growth and yield under low and high NO_3_- supply	
9	*OsNPF6.1HapB*	Rice	Root cells	Transactivation of OsNPF6.1HapB by OsNAC42	• Improves N uptake and signaling pathway under N starvation	(Tang et al., 2019)
					• Improves NUE and yield	
10	*OsNRT1.1A* (*OsNPF6.3)*	Rice	Epidermis, Root vascular tissues, parenchyma cells of both culms and leaf sheaths	CaMV 35S promoter	• Enhances N-utilization and flowering, and grain yield	(Wang et al., 2018c)
					• Shortens maturation time	
					• Increases the expression of N-utilization and flowering-related genes.	
11	*OsNPF2.4*	Rice	Root epidermis, phloem companion cells, and xylem parenchyma	Ubiquitin promoter	Enhances N acquisition and long-distance transport	(Xia et al., 2015)
13	OsNPF2.2	Rice	Leaves and branches	OsNPF2.2 promoter-β-glucuronidase	Affects root-to-shoot ${\text{NO}}_{3^{-}}$ transport and plant growth.	(Li et al., 2015)
14	*LeNRT2.3*	Tomato	Rhizodermal and pericycle cells in roots.	CaMV 35S promoter	Enhances ${\text{NO}}_{3^{-}}$ uptake, and transport to the shoot	(Fu et al., 2015)
15	*NRT2.7*	Arabidopsis	Seeds and siliques	CaMV 35S promoter	Regulates nitrate content in mature seeds	(David et al., 2014)
16	NPF3	Arabidopsis	Root epidermis	CaMV 35S promoter	Partly regulates gibberellin distribution	(Tal et al., 2016
17	*OsNPF7.9*	Rice	Xylem parenchyma cells	CaMV 35S promoter	Regulates ${\text{NO}}_{3^{-}}$ allocation	(Guan et al., 2022)
					Coordinates growth and stress tolerance	
18	*OsNPF5.16*	Rice	Roots, leaf sheaths, and tiller basal parts	Ubiquitin promoter	Improves sheath ${\text{NO}}_{3^{-}}$ content, tiller number, and biomass	(Wang et al., 2022)
19	*OsNPF3.1*	Rice	Culms, panicle and, aerial parts of the roots	pYLCRISPR/Cas9 vector	• Enhances NUE	(Yang et al., 2023)
					• May participate in shoot N allocation	
20	*MeNPF4.5*	Cassava	Root	CaMV35S promoter	• Regulates N uptake and utilization, thus improving NUE in cassava.	(Liang et al., 2022)
					• Improves photosynthesis and N-enzymatic activities.	

The expression of several NRT2 transporters has also been found to influence yield and NUE under N-starved conditions. *NRT2.2* was upregulated to improve N uptake, assimilation, and plant growth under low ${\text{NO}}_{3^{-}}$ conditions (Li et al., 2007). Under the same ${\text{NO}}_{3}^{–}$ stressed conditions, *TaNRT2.5*, highly expressed in wheat, increases ${\text{NO}}_{3^{-}}$ uptake and root growth (Guo et al., 2014). Chen et al. (2016) conducted a study on transgenic rice and observed that *OsNRT2.1*, which has the OsNAR2.1 promoter (*pOsNAR2.1: OsNRT2.1*), was upregulated in the roots and culms. This upregulation significantly increases the overall yield, biomass, and NUE in transgenic lines harboring *OsNAR2.1* (*pOsNAR2.1: OsNRT2.1*). However, the reverse (decrease in NUE) was obtained with the constitutive promoter of OsNRT2.1 (*pUbi: OsNRT2.1*). These variations could be accrued to alterations in the localization and abundance of *OsNRT2.1* in the plant tissue (Chen et al., 2016). Further investigations regarding the importance of the NRT2 gene in NUE showed that two variants, *OsNRT2.3a* and *OsNRT2.3b*, were identified in rice. The elevated expression of *OsNRT2.3b* enhances intracellular pH balance under the synergetic supply of ${\text{NH}}_{4}^{+}$ and ${\text{NO}}_{3^{-}}$ , thereby increasing the uptake capacity of other nutrients (P, N, and Fe) and ultimately increasing grain yield and NUE by 40% (Fan et al., 2016b). This result demonstrates the importance of pH sensing by *OsNRT2.3b* in improving plant NUE and adaptation of rice to changes due to different ${\text{NH}}_{4}^{+}$ **-** ${\text{NO}}_{3^{-}}$ supplies. However, this N uptake and transport function observed in *OsNRT2.3b* was lost in *OsNRT2.3a* (Fan et al., 2016b; Chen et al., 2020a). *OsNRT2.3a* cannot independently improve crop yield and NUE due to its inability to increase the expression of *OsNAR2.1* (Chen et al., 2020a). Thus, the coexpression of *OsNRT2.3a* with the OsNAR2.1 promoter becomes imperative to enhance rice N use. The literature reviewed thus far has demonstrated a need for most NRT family members to be coexpressed with specific promoters to effectively enhance plant growth, biomass, and NUE, especially in Arabidopsis and rice; however less in known in other crop species.

## 6 Nitrate transporters and environmental cues: Influence of environmental stress factors and inducers on nitrate allocation to roots

Numerous studies have investigated the crucial roles of ${\text{NO}}_{3^{-}}$ transporters in mediating the uptake and long**-**distance transport of ${\text{NO}}_{3^{-}}$ ; however, less is known towards understanding transport systems involved in ${\text{NO}}_{3^{-}}$ reallocation under biotic and abiotic stresses. ${\text{NO}}_{3^{-}}$ transporters play crucial roles in the plants’ response to adverse environmental conditions. Indeed, plants acclimatize better to environmental stress when less ${\text{NO}}_{3^{-}}$ is allocated to the shoot. Thus, this section examines the contribution of ${\text{NO}}_{3^{-}}$ transporters in assisting plants to strive in adverse environmental conditions.

**Figure 2:**
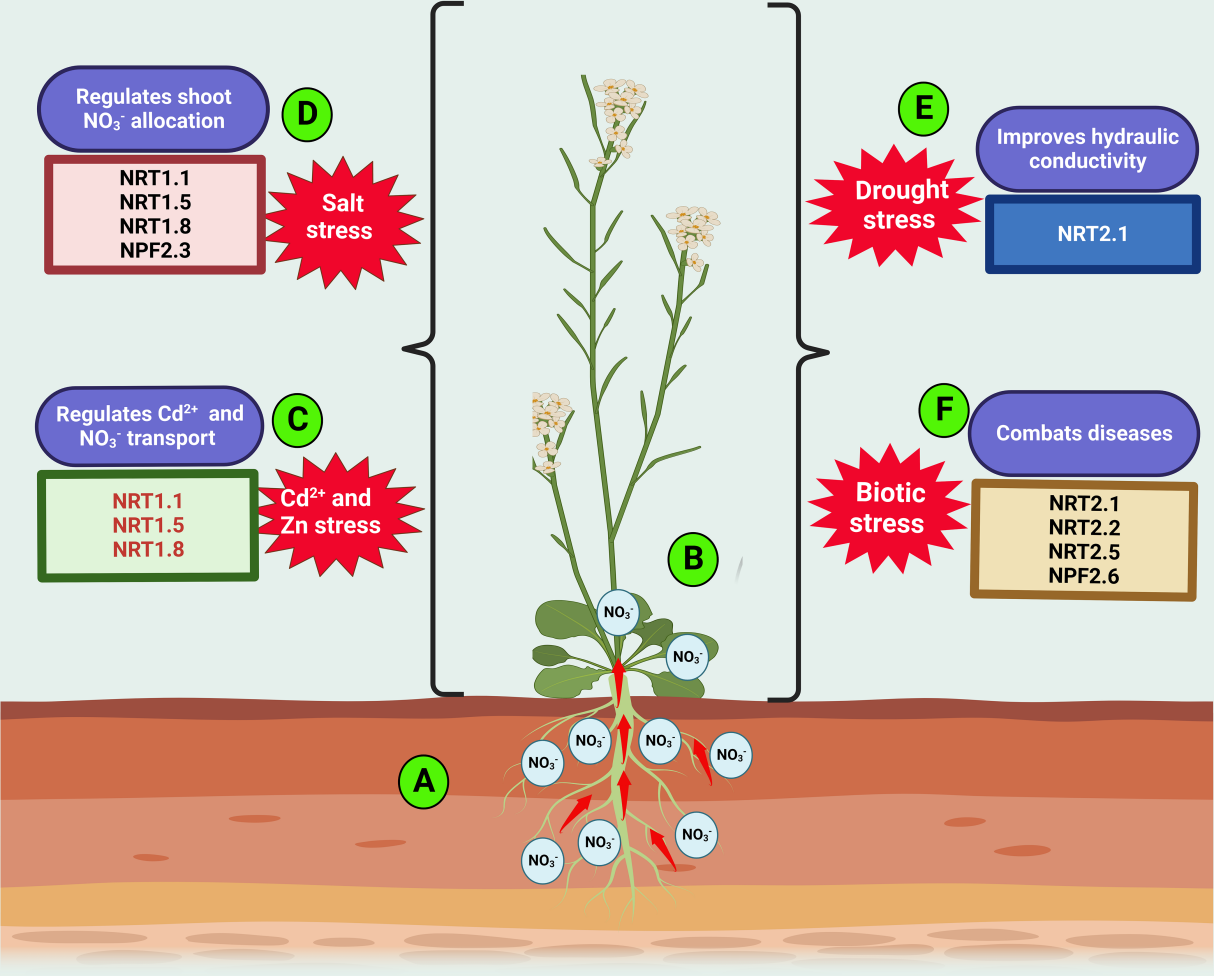
Roles of nitrate transporters in plant response to adverse environmental conditions. Environmental cues including heavy metals (Cd^2+^ and Zn), salinity, drought, and pathogenic stress engender reduction in plant growth and NUE. The resulting stressed plants accumulate more ${\text{NO}}_{3^{-}}$ at the root **(A)** while retaining less in the shoot **(B)**. Under Cd^2+^ or Zn stress, nitrate transporters, *NRT1.1*, *NRT1.5* and *NRT1.8* concurrently regulates Cd^2+^ or Zn uptake and ${\text{NO}}_{3^{-}}$ allocation to the root **(C)**. The transporters involved in root-to-shoot allocation of ${\text{NO}}_{3^{-}}$ under salinity include *NPF2.3*, *NRT1.1*, *NRT1.5*, and *NRT1.8* **(D)**. *NRT2.1* promotes plants’ tolerance to drought stress **(E)**. In addition to *NRT2.1*, *NRT2.2*, *NRT2.5* and *NRT2.6* are involved in biotic stress regulation **(F)**.

The quantity of ${\text{NO}}_{3^{-}}$ translocated from roots to shoots varies under diverse environmental conditions, as this could positively or negatively affect plant NUE. Hence, ${\text{NO}}_{3^{-}}$ redistribution in plants is a prerequisite to improved plant growth under N shortages and adverse conditions (Fan et al., 2017). Stressed plants tend to uptake and transport less ${\text{NO}}_{3^{-}}$ to the shoot while retaining more nitrate in its root than required (**Figure 2**). Such ${\text{NO}}_{3^{-}}$ allocation to the root as induced by environmental fluctuations (including biotic and abiotic stress) is referred to as “stress-initiated nitrate allocation to roots” (SINAR) (Zhang et al., 2018). Over two decades ago, Hernandez et al. (1997) investigated the inherent effects of cadmium (Cd^2+^) on ${\text{NO}}_{3^{-}}$ uptake, and distribution in pea plants. They found that ${\text{NO}}_{3^{-}}$ was increasingly retained at the plant root, and fewer ${\text{NO}}_{3^{-}}$ were reallocated to the shoot of Cd-treated pea compared with the control, thereby disrupting the NUE of plants (**Figure 2**). However, the study could not elucidate the mechanism underlying the fluctuation in the root-to-shoot transport of ${\text{NO}}_{3^{-}}$ . Many years later, several research investigations have shown the active involvement of ${\text{NO}}_{3^{-}}$ transporters in regulating Cd^2+^ uptake and other SINAR**-**related stress conditions (Lin et al., 2008; Zhang et al., 2014). Mao et al. (2014) reported *NRT1.1* as a potential regulator of Cd^2+^ uptake in plants. They observed that plants exposed to Cd^2+^ stress exhibit repression of *NRT1.1* and, as such, exert a negative influence on plant N nutrition (**Figure 2**). Thus, the loss of *NRT1.1* function reduced Cd^2+^ in the roots and shoots, improving plant biomass production under Cd^2+^ stress (**Figure 2**). Although the disruption of *NRT1.1* activity induced by Cd^2+^ stress negates ${\text{NO}}_{3^{-}}$ uptake, it enhances plant tolerance to Cd^2+^ stress by reducing Cd^2+^ influx into the root. A recent study by Jian et al. (2019) opined that overexpression of NRG2 (which functions downstream of *NRT1.1*) in wild-type and *nrt1.1* increased root ${\text{NO}}_{3^{-}}$ over shoot nitrate, thus alleviating Cd^2+^ toxicity. These findings demonstrate the involvement of *NRT1.1* in regulating cadmium uptake while coordinating nitrate allocation to the root. *NRT1.1* also regulates Zn accumulation in Arabidopsis by improving ${\text{NO}}_{3^{-}}$ uptake in the wild type through a ${\text{NO}}_{3}^{–}$ dependent pathway under Zn stress (**Figure 2**) (Pan et al., 2020).

In addition to *NRT1.1*, *NRT1.5* and *NRT1.8* regulate the acropetal reallocation of ${\text{NO}}_{3^{-}}$ to shoots under cadmium and salinity stress (Fan et al., 2017a). Such stresses activate antagonistic expression of the two latter genes (*NRT1.5* and *NRT1.8*), with reduced expression of *NRT1.5/NPF7.3* (Chen et al., 2012) and increased expression of *NRT1.8/NPF7.2* (**Figure 2**) (Li et al., 2010). From the study conducted by Li et al. (2010), loss of *NRT1.8* function displays greater sensitivity to Cd^2+^ stress than wild-type plants under high ${\text{NO}}_{3^{-}}$ conditions. However, an opposite effect was observed, with *nrt1.5* mutants having greater Cd^2+^ tolerance in relation to the control. The Cd^2+^ sensitivity observed with the *ntr1.8* mutants could be due to Cd^2+^ translocation to its shoots, thus counteracting the plant adaptive strategy that supports Cd^2+^ accumulation in plant roots. The upregulation of *NRT1.8* expression triggers nitrate removal from the xylem under Cd^2+^-stressed conditions. This result suggests a strong link between Cd^2+^ tolerance and ${\text{NO}}_{3^{-}}$ allocation.

In addition to *NRT1.5* and *NRT1.8*, *NPF2.3* also contributes to the SINAR response under salt stress. Nitrate allocation to the shoot was drastically reduced under salt-stressed conditions due to the unaltered expression of *NPF2.3* and partial expression of the *NPF7.3* gene in the root stele. However, the loss of *NPF2.3* function led to the reduced root**-**to**-**shoot allocation of ${\text{NO}}_{3^{-}}$ (**Figure 2**) (Taochy et al., 2015). These data demonstrate the quantitative and physiological contribution of the ${\text{NO}}_{3^{-}}$ efflux transporter *NPF2.3* to ${\text{NO}}_{3^{-}}$ allocation to the shoot under salinity (Taochy et al., 2015; Chao et al., 2021). Alvarez-Aragon and Rodriguez-Navarro (2017) also found Na^+^ accumulation to be partially defective in the *nrt1.1* mutant, demonstrating the partial contribution of *NRT1.1* to ${\text{NO}}_{3}^{–}$ dependent Na^+^ transport (**Figure 2**). Plants expressing these ${\text{NO}}_{3}^{–}$ related genes in response to heavy metal or salt stress exhibit enhanced ${\text{NO}}_{3^{-}}$ uptake, plant growth, and tolerance to heavy metal- or salt-stressed environments.

Previous physiological research investigations have shown varying impacts of ${\text{NO}}_{3^{-}}$ and ${\text{NH}}_{4}^{+}$ availability on water uptake and transport in plants subjected to water stress (Guo et al., 2007). They found that the assimilation rate and stomatal conductance of ${\text{NH}}_{4}^{+}$ **-**fed plants surpassed those of NO_3_^-^-fed plants; thus, ${\text{NH}}_{4}^{+}$ nutrition improves rice seedling tolerance to drought (Guo et al., 2007). Li et al. (2016a) revealed that the high-affinity NO_3_- transporter *NRT2.1* alters ${\text{NO}}_{3^{-}}$ accumulation to regulate root hydraulic conductivity (**Figure 2**). They found *NRT2.1* to be a positive regulator of plasma membrane intrinsic protein PIPs. This latter study unraveled the link between ${\text{NO}}_{3}^{-}$ use, water stress, and *NRT2.1* expression, indicating the potential roles of *NRT2.1* in drought tolerance (Li et al., 2016a). However, a more recent investigation has shown how the high**-**affinity ${\text{NO}}_{3^{-}}$ transporter partner protein *OsNAR2.1* positively regulates drought-related responses to stress and enhances drought tolerance in rice (**Figure 2**) (Chen et al., 2019).

Ample agronomic evidence exists regarding the impact of excessive N fertilizer use on the incidence rate of plant diseases (Fagard et al., 2014; Fan et al., 2017). For example, excessive N fertilizer application triggers the severity of powdery mildew caused by a biotrophic pathogen that saps plant nutrients. Interestingly, a reduction in N fertilizer application has been found to reduce Arabidopsis tolerance to *Erwinia amylovora*. These findings indicate a complex relationship between N uptake, metabolism, and disease infection processes. Thus, it is evident that N status affects plant tolerance or susceptibility to diseases under specific environmental conditions (Fagard et al., 2014). Unfortunately, the molecular mechanism underlying the impact of ${\text{NO}}_{3^{-}}$ transporters on fungal infection or pathogenic attack is not fully understood. To investigate the possible mechanisms involved in N uptake by the biotrophic pathogen, Pike et al. (2014) characterized the low**-**affinity transporter *VvNPF3.2* (in grapevine) and cloned Arabidopsis ortholog *NPF3.1*. In this study, powdery mildew pathogen infection was shown to upregulate the expression of *VvNPF3.2* and *NPF3.1* in vascular tissues, major and minor veins of leaves. The loss of *NRT2.1* and *NRT2.2* under N**-**deficient conditions resulted in increased resistance to *Pseudomonas syringae* pv tomato DC3000 infection (**Figure 2**) (Li et al., 2007; Camanes et al., 2012). Additionally, in the NRT2 family, the roles of two putative high-affinity ${\text{NO}}_{3^{-}}$ transporters, *NRT2.5* and *NRT2.6*, were investigated in response to rhizospheric bacterium STM196 using single and double Arabidopsis mutants (Kechid et al., 2013). The study revealed that mutations in *NRT2.5* and *NRT2.6* inhibited plant growth and abolished root system architecture in response to STM196. Hence, Arabidopsis leaves expressing *NRT2.5* and *NRT2.6* appear to play crucial roles in the plant response to STM196 in a ${\text{NO}}_{3^{-}}$ uptake-independent manner (**Figure 2**). The expression of both genes (*NRT2.5* and *NRT2.6*) is also crucial for promoting plant growth mediated by STM196 (Kechid et al., 2013). Recently, T**-**DNA mutants of *NRT2.5* showed stronger resistance to *Pseudomonas syringae* pv. tomato DC3000 inoculation compared to its wild-type counterpart, an indication of *NRT2.5* role in plant biotic defense (Du Toit et al., 2020; Devanna et al., 2021). These research findings have demonstrated the functional roles of ${\text{NO}}_{3}^{-}$ transporters in the plant response to biotic stress, while suggesting safe, innovative, and sustainable means of controlling crop pathogens.Mycorrhizal colonization of rice root also appears to promote the expression of a putative nitrate transporter, *OsNPF4.5*. This result improved growth and yield properties in host plant (Wang et al., 2020c). However, inactivation of *OsNPF4.5* resulted in the reduction of arbuscule incidence, as well as a depletion in symbiotic nitrogen uptake in rice (Wang et al., 2020c).

Another member of the nitrate and peptide transporters family (NPF), *OsNPF8.1* (*OsPTR7*), a putative peptide transporter in rice (localized in the cell plasma membrane), has been reported as permeable to methylated arsenic species, especially, dimethylarsenate (DMA). *OsNPF8.1* is involved in long-distance transport of arsenic in rice (Tang et al., 2017). However, the peptide-mediated transport of arsenic species has been linked with imbalance nutrient (especially, phosphate) supply in plants (Finnegan and Chen, 2012). Consequently, it is imperative to investigate the activity of *OsNPF8.1* on N uptake, as well as the collateral accumulation of DMA, its clinical significance and nutrient imbalance in economically significant crops.

## 7 Could nitrate uptake and utilization affect the efficiency of other plant nutrients?

Balanced nutrition is paramount to maintaining good human health, and this is achievable by eating a balanced diet. In plants, maintaining an appropriate nutrient balance is also required because excessive accumulation of a specific nutrient might affect the uptake of the other and vice versa (Aluko et al., 2021). This nutritional balance ultimately affects crop growth and plant nutrient use efficiency (Bouain et al., 2019). Such nutritional crosstalk coexists between phosphorus (P) and N, the most limiting nutrient element required for crop growth and development. Phosphorus starvation reduces nitrate uptake capacity in tobacco (Rufty et al., 1990), maize (De Magalhães et al., 1998), and barley (Lee, 1982). These phenomena demonstrate the mechanisms involved in optimizing nutrient uptake and utilization to maintain plant homeostatic balance. Molecular evidence indicates that nitrogen limitation adaptation (NLA) ubiquitin offsets ${\text{NO}}_{3^{-}}$ deficiency induced by excessive P *via* degradation of *PHT1*, the phosphate transporter (Kant et al., 2011b). The phenotypic analysis illustrated the functional role of nitrate**-**inducible garp**-**type transcriptional repressor 1.2 (*NIGT1.2*) in integrating N and P signals. Under sufficient P supply, *NIGT1.2* was not activated due to the coexpression of *PHR1* and SPXs, which are P**-**sensor proteins and repressors of *PHR1*, respectively (Medici et al., 2015). However, *PHR1* was detached from the inhibitors SPX1/2/3/4 to promote the expression of NIGT1 clade genes under P**-**starved conditions. Thus, nitrate uptake is suppressed due to P deficiency through the PHR1-NIGT1**-**NRT2.1 pathway (Maeda et al., 2018). With such development, N uptake regulation *via* the PHR1**-**NIGT1 path could be a good adaptative mechanism under P starvation (Maeda et al., 2018). Another recent study found that *NIGT1.2* increased the expression of phosphate transporters (PHT1;1 and PHT1;4) but repressed the nitrate transporter *NRT1.1*, an indication that *NIGT1.2* could maintain a balance between N and P to improve N uptake and utilization under (phosphorus) P starvation (Wang et al., 2020b).

The highly ${\text{NO}}_{3}^{–}$ inducible NRT1.1**-**controlled GARP transcription factor, *HRS1*, and its closest homolog, *HHO1*, function downstream of NRT1.1, NLP6, and NLP7. However, *HRS1* and *HHO1* act as major primary root growth inhibitors only when the media is P**-**starved in the presence of ${\text{NO}}_{3^{-}}$ , indicating extensive integration of the N and P signaling networks (Medici et al., 2015). Following the previous discussion on how HRS1 mediates N and P crosstalk, Medici et al. (2019) found that PSR marker gene responses depend on the N supplied. Indeed, transcript levels of *PHO2* were coordinated by nitrate availability accumulated during both high and low supplies of nitrate. Notably, this nitrate-induced strategy of PSR regulation is conserved in plants. However, several *PSR* genes were not regulated by ${\text{NO}}_{3^{-}}$ in a *pho2* mutant, indicating that *PHO2* incorporates nitrate signals into PSR (Medici et al., 2019). Upon P starvation, *NRT1.1* is downregulated, while *PHO2* functions to positively regulate *NRT1.1*. In rice, the genes induced by P starvation *OsIPS1*, *OsSPX1*, and the P transporter *OsPT1* only respond to P starvation when nitrate is present (Medici et al., 2019). On the overall assessment, these findings elucidate the complexity of nitrate and phosphorus responses while emphasizing the principal roles of *NRT1.1* in regulating the interaction.

Another macronutrient required for plant health is potassium (K^+^), as it strongly coordinates nitrate ( ${\text{NO}}_{3^{-}}$ ). Previous reports indicated that *NRT1.5* facilitates the long-distance transport of ${\text{NO}}_{3^{-}}$ and K^+^ in a nitrate**-**dependent manner (Meng et al., 2016; Zheng et al., 2016). *NRT1.5*, expressed in the pericycle of root cells, participates in the xylem loading of nitrate. When there is a K deficit, *NRT1.5* directly triggers the movement of K^+^ to the root xylem for root**-**to**-**shoot transport. This investigation demonstrates the crucial role of *NRT1.5* in root-to-shoot K^+^ transport and its involvement in the synergetic regulation of ${\text{NO}}_{3^{-}}$ /K^+^ distribution in plants (Li et al., 2017b). Another study reported that MYB59 activates the expression of *NRT1.5* and binds directly to its promoter to ensure a controlled nutrient distribution from root to shoot. When plants become deficient in ${\text{NO}}_{3^{-}}$ /K^+^, the expression of MYB59 and *NRT1.5* is repressed to maintain a balanced ${\text{NO}}_{3^{-}}$ /K^+^ distribution between the roots and shoots (Du et al., 2019).

## 8 Nitrate transporter regulates nitrate and auxin crosstalk for root growth and nitrogen uptake

Evidence has shown the impact of changes in N status on auxin distribution in plants (Hou et al., 2021). Compared with moderate N supply, limited ${\text{NO}}_{3^{-}}$ supply engenders auxin deposition in the roots of Arabidopsis, wheat, soybean, maize, and rapeseed (Caba et al., 2000; Tian et al., 2008; Asim et al., 2020), indicating the importance of *in situ* auxin synthesis in the root (Yang et al., 2022). Thus, the *in situ* auxin synthesis and the shoot-to-root polar transport jointly contributes to auxin deposition in the root under N limitation (Yang et al., 2022). In contrast, a 30% reduction in root indole-3-acetic acid (the putative among natural auxins) content was observed when the amount of ${\text{NO}}_{3^{-}}$ supplied to rice dropped from 2.5mM to 0.01mM (Sun et al., 2014b). Perhaps, the discrepancies in N induced auxin response stems from varying plant growth conditions and the species involved. Nevertheless, all these findings demonstrate the importance of nitrate and auxin crosstalk in root development, and the mechanism of such responses are triggered by the activities of ${\text{NO}}_{3^{-}}$ transporters.

In addition to the ${\text{NO}}_{3^{-}}$ transport and signaling function, *NRT1.1*, among other transporters, facilitates basipetal transport of auxin and negatively regulates auxin biosynthetic genes, *TAR2* and *LAX3*, under ${\text{NO}}_{3^{-}}$ deficiency (Maghiaoui et al., 2020). As a consequence, *NRT1.1* removes auxin (required for lateral root growth) deposited at the lateral root primordia, inhibiting lateral root growth under such condition. All these inhibitory effects of *NRT1.1*, including root growth reduction and patchy auxins are alleviated in response to high ${\text{NO}}_{3^{-}}$ supply (Maghiaoui et al., 2020). Thus, *NRT1.1*-mediated auxin transport was disrupted and its (*NRT 1.1*) expression repressed, to facilitate lateral root growth and auxin accumulation at the root tip under increasing ${\text{NO}}_{3^{-}}$ supply (Remans et al., 2006). These findings indicated that *NRT1.1* functions in reprogramming root system architecture in response to ${\text{NO}}_{3^{-}}$ availability. However, the integrated function of this molecular circuit is yet unraveled.

Although, it is understood that external N status regulates auxin biosynthetic genes and signaling pathways. However, less is known about the identities of auxin-related genes that are N-responsive, and whether these genes reprogram plant N metabolism to improve crop NUE is yet unexplored. To this end, Zhang et al. (2021b) identified DULL NITROGEN RESPONSE1 (DNR1) as an intriguing QTL regulating auxin and N crosstalk for NUE improvement in rice. DNR1 mediates plant N metabolism by counteracting the auxin deposited in response to N availability. This process enhances auxin biosynthesis and induces AUXIN RESPONSE FACTOR, a major regulator of N-responsive genes to improve NUE and grain yield.

Out of the identified ${\text{NO}}_{3^{-}}$ transporters, the functions of the ${\text{NO}}_{3}^{–}$ transceptor’s (*NRT1.1*) in auxin regulation has been the most investigated. However, less is known about the versatile functions of other ${\text{NO}}_{3}^{–}$ related proteins in regulating other plant developmental traits.

## 9 Integrated approaches to improve plant NUE

Genetic modification of crops has been a promising strategy for improving plant N use through diverse breeding techniques during the past few decades. Indeed, several ${\text{NO}}_{3^{-}}$ transporter genes, their regulators, and other ${\text{NO}}_{3^{-}}$ responsive genes regulating NUE have been well studied. However, mechanisms involved in this regulation, which specifically describes the strategies involved in NUE improvement, have been overlooked due to difficulties in identifying N-specific phenotypes. (Hu et al., 2015) revealed that genetic variation of the major quantitative trait locus (QTL) *NRT1.1B* (*OsNPF6.8*) promotes NUE divergence between *Indica* and *Japonica* rice subspecies. They found that *NRT1.1B* from indica improved the tiller number, NUE, and grain yield of *Japonica* rice. Several other QTL-based approaches have generated signaling proteins, transcriptional regulators, and components of hormonal pathways that regulate plant NUE. One of these is a QTL study that used positional cloning and genetic complementation to map out DEP1 (Dense and erect panicles 1), a heterotrimeric G protein that confers a significant yield increase (Sun et al., 2014a). Under moderate N fertilization, plants harboring the dominant allele *DEP1-1* display N-insensitive vegetative growth, as well as improved N uptake and assimilation, thereby increasing yield (Sun et al., 2014a). This result implies that modulating the activity of DEP1 could provide a lasting strategy for grain yield increases in rice. Another QTL study showed that the accumulation of the growth inhibitor DELLA confers semi-dwarfism and reduces NUE in rice (Li et al., 2018). However, the NUE and grain yield of green revolution varieties are restored by tilting the GRF4–DELLA stability toward an increased abundance of GRF4. This study indicated that regulating physiological activities and plant growth induced by efficient N use could open up innovative breeding ideas for sustainable food security (Li et al., 2018). Although QTL analysis has also informed the recent NUE gene identification strategy in crop species such as maize (Zhang et al., 2019), the importance of QTL analysis is yet unknown in some other higher plants.

In addition to QTL analysis, other analytical studies involving genome-wide association studies (GWAS) could be used to identify an array of NUE candidate genes in Arabidopsis (Atwell et al., 2010), maize (Li et al., 2013), rice (Si et al., 2016), and other crop species (Korte and Farlow, 2013; Ogura and Busch, 2015). An elite haplotype of the nitrate transporter *OsNPF6.1HapB* was recently identified using GWAS (Tang et al., 2019). This allele improved nitrate uptake, NUE, and grain yield under N-deficient conditions. In the same study, the NUE-related transcription factor OsNAC4 was used to transactivate *OsNPF6.1^HapB^*, thereby increasing plant NUE and grain yield. This result suggests that the NAC42-NPF6.1 signaling cascade is a promising strategy for improving NUE and rice yield (Tang et al., 2019).

To further identify the genes enhancing NUE, Clustered Regularly Interspaced Palindromic Repeats (CRISPR)/Cas9 along with the Cas9 nuclease (CRISPR/CAS9) system was developed. CRISPR/CAS9 has been deployed to facilitate easy and robust technology to edit genes for improved plant N use. Multiple applications of CRISPR/CAS9 technology have been demonstrated in major crops, including sorghum, rice, and tomatoes (Ito et al., 2015; Ma et al., 2015). Notably, CRISPR/CAS9 mostly mutates negative growth regulators instead of overexpressing positive regulators, thereby providing prospects for crop breeding (Tiwari et al., 2020). A related strategy described one of the Bric**-**a**-**Brac/Tramtrack/Broad gene family members, *BT2*, that downregulates the *NRT2.1* and *NRT2.4* genes (Araus et al., 2016), thus reducing ${\text{NO}}_{3^{-}}$ uptake and NUE under low ${\text{NO}}_{3^{-}}$ conditions. When this *BT2* gene was mutated in Arabidopsis, a 65% increment in nitrate uptake was observed, while mutation of *OsBT2* yielded a 20% increase in NUE compared to wild-type under poor ${\text{NO}}_{3^{-}}$ supply (Araus et al., 2016). To date, the functions and features of a significant number of negative regulators or inhibitors of nitrate transporters have yet to be functionally characterized in plants. Hence, it is plausible that gene editing or mutating their expression by CRISPR/Cas9 appears to be a promising strategy for achieving future breeding goals (Tiwari et al., 2020).

It is essential to note that incorporating transcriptomics, proteomics, and metabolomics, which characterize the expression profile, could facilitate the identification of agronomically induced genes or pathways. Moreover, computational and system biology could aid in identifying candidate genes during domestication.

## 10 Conclusion and future perspectives

Nitrate transporters have not only been shown to function in plant uptake and transport capacity; their vital roles and potential in improving plant N use have also guaranteed the possibility of meeting future global food demands. Indeed, improved ${\text{NO}}_{3^{-}}$ uptake and utilization ( ${\text{NO}}_{3^{-}}$ transport, remobilization, and assimilation) through transporter activity is a prerequisite to attaining increased NUE and overall plant growth. With the understanding that the activities of these ${\text{NO}}_{3^{-}}$ transporters are enhanced when co-expressed with their specific promoters or Tfs, it becomes imperative to select and integrate NO_3_ **^–^** specific promoters with their transporters for efficient plant N utilization. An excellent way to improve ${\text{NO}}_{3^{-}}$ utilization could be to carefully select senescence-specific promoters (primarily expressed in source organs or leaves) to facilitate phloem-expressed nitrate transporters. Most research works have successfully established the impact of nitrate transporters on adverse environmental conditions (biotic and abiotic stress). They have also addressed their relationships with other plant nutrients only under controlled conditions; however, field-based studies affirming these functions are scarce.

Moreover, relatively few ${\text{NO}}_{3^{-}}$ transporters performing complex interplay functions have been identified, while the established ones were found to play multiple physiological roles in environmental and nutritional stresses. The underlying mechanisms behind these multipurpose functions are unknown, and the extent to which these transporters can mitigate abiotic stress is unresolved. Thus, to understand and manipulate the functional roles of nitrate transporters in enhancing plant NUE under diverse conditions, future research should address some critical questions, including the following, but not limited to:

- How do the combined effects of biotic/abiotic stressors influence nitrate transporter activities, and to what extent?
- Does the uptake of other macro- and micronutrients alter the expression or impair the prospective function of nitrate transporters and vice versa?
- Is there a possibility of having nutrient imbalance feedback due to alterations in the expression of either nitrate transporters or the transporters of other nutrients (macro- and micronutrients)?
- If the activities of nitrate transporters are eventually established to significantly affect the uptake of other nutrients and vice versa, what molecular techniques could be factored in to recuperate such imbalance?
- Could the crosstalk between N-responsive and auxin biosynthesis genes affect the uptake of other essential nutrients by plants?
- Could specific ${\text{NO}}_{3^{-}}$ transporters or related genes function or be expressed differently in diverse crop species?
- Could models be developed to project or predict the possible influence of biotic and abiotic environmental parameters, as well as their complex interplay on the NUE of individual plant species?

Developing profound resolutions to these questions will afford us a better understanding of how nitrate transporters could be maximized to enhance plant NUE under adverse environmental conditions. Knowledge of these factors will also help settle crises related to plant nutritional imbalance and cross-talk, thereby achieving plant breeding goals for quality and sustainable food production.

## Author contributions

Conceptualization, OOA, QW, and HL; writing-original draft, OOA; review and editing; SK and OMA; visualization, OOA, GY, and CL; validation, SK, OMA, QW, and HL; supervision, QW and HL; funding acquisition, QW and HL. All authors contributed to the article and approved the submitted version.
